# Supplementary material for: Effects of partial penectomy for penile cancer on sexual function: A systematic review
Source: PLoS One. 2022 Sep 22;17(9):e0274914. doi: 10.1371/journal.pone.0274914 (PMC9499284; doi:10.1371/journal.pone.0274914)
Supplement: S1 File — (DOCX) [file pone.0274914.s002.docx]

**Supplementary material 1**

**PubMed**

((“sexual behavior"[MeSH Terms] OR "sexual dysfunctions, psychological"[MeSH Terms] OR "sexual dysfunction, physiological"[MeSH Terms] OR "penile erection"[MeSH Terms] OR "erectile dysfunction"[MeSH Terms] OR ("orgasm"[MeSH Terms] and “function*”[All Fields]) OR "libido"[MeSH Terms] OR "quality of life"[MeSH Terms] OR “quality of life”[All Fields]) OR ((“sexual”[All Fields] OR “erectile”[All Fields] OR “orgasmic”[All Fields] OR “intercourse”[All Fields] OR “sex”[All Fields]) AND (“function*”[All Fields] OR “dysfunction*”[All Fields] OR “desire”[All Fields] OR “satisfaction”[All Fields] OR “libido”[All Fields] OR “drive”[All Fields]))) AND (“penectomy” OR “phallectomy”)

**The Cochrane Library**

#1. MeSH descriptor: [Sexual Behavior] explode all trees

#2. MeSH descriptor: [Sexual Dysfunctions, Psychological] explode all trees

#3. MeSH descriptor: [Sexual Dysfunction, Physiological] explode all trees

#4. MeSH descriptor: [Penile Erection] explode all trees

#5. MeSH descriptor: [Erectile Dysfunction] explode all trees

#6. MeSH descriptor: [Orgasm] explode all trees

#7. "function"

#8. MeSH descriptor: [Libido] explode all trees

#9. MeSH descriptor: [Quality of Life] explode all trees

#10. "quality of life"

#11. "sexual"

#12. "erectile"

#13. "orgasmic"

#14. "intercourse"

#15. "sex"

#16. "function*"

#17. "dysfunction*"

#18. "desire"

#19. "satisfaction"

#20. "libido"

#21. "drive"

#22. "penectomy"

#23. "phallectomy"

#24. ((#1 OR #2 OR #3 OR #4 OR #5 OR (#6 AND #7) OR #8 OR #9 OR #10) OR ((#11 OR #12 OR #13 OR #14 OR #15) AND (#16 OR #17 OR #18 OR #19 OR #20 OR #21))) AND (#22 OR #23)

**EBSCO – (MEDLINE, CINAHL, Open Dissertations)**

S1. (MH "sexual behaviour")

S2. (MH "sexual dysfunctions, psychological")

S3. (MH "sexual dysfunction, physiological")

S4. (MH "penile erection")

S5. (MH "erectile dysfunction")

S6. (MH "orgasm")

S7. "function*"

S8. (MH "libido")

S9. (MH "quality of life")

S10. "quality of life"

S11. "sexual"

S12. "erectile"

S13. "orgasmic"

S14. "intercourse"

S15. "sex"

S16. "function*"

S17. "dysfunction*"

S18. "desire"

S19. "satisfaction"

S20. "libido"

S21. "drive"

S22. "penectomy"

S23. "phallectomy"

S24. ((S1 OR S2 OR S3 OR S4 OR S5 OR (S6 AND S7) OR S8 OR S9 OR S10) OR ((S11 OR S12 OR S13 OR S14 OR S15) AND (S16 OR S17 OR S18 OR S19 OR S20 OR S21))) AND (S22 OR S23)

**Open Grey**

(quality of life OR (sexual OR erectile OR orgasmic OR intercourse OR sex) AND (function* OR dysfunction* OR desire OR satisfaction OR libido OR drive)) AND (penectomy OR phallectomy)
