# Supplementary material for: Effects of partial penectomy for penile cancer on sexual function: A systematic review
Source: PLoS One. 2022 Sep 22;17(9):e0274914. doi: 10.1371/journal.pone.0274914 (PMC9499284; doi:10.1371/journal.pone.0274914)
Supplement: Attachment — Submitted filename: Response to reviewers.docx [file pone.0274914.s003.docx]

**REVIEWER #1 COMMENTS**

**Reviewer comment 1:**

I do understand that you applied a narrative (descriptive) synthesis of the literature, but please refrain from using ambiguous statements like "was broadly comparable across the four studies".

**Author response:**

Thank you very much for this suggestion. We have re-read the text and made adjustments to avoid use of ambiguous language. In particular, for the sentence highlighted above around line 390-391, we have adjusted the text to the following, more specific language:

**‘***This suggests that despite the different surgical techniques, pre-operative sexual function scores, and remaining penile length, sexual function following partial penectomy was similar (i.e., the difference in scores was < 3 points between studies for most IIEF domains)* *across the four studies.*’

**Reviewer comment 2:**

You highlighted some very important limitations of the study, but I do appreciate the importance of your research. Your research question(s) is clear, your results based on the applied method is supported and the manuscript is presented in an understandable and concise manner.

**Author response:**

Thank you very much, we are grateful for your feedback.

**EDITORIAL COMMENTS**

**Editorial comment 1:**

The manuscript has been well written. Given 4 studies were identified and included in the systematic review. An updated search may help identify additional articles published after May 2021 and would enrich the content of the manuscript.

**Author response:**

Thank you for your comment and for taking the time to appraise our manuscript. We have now re-run the searches (on 24^th^ March, 2022) in the hope of identifying additional relevant research. We identified an additional 25 articles. Following removal of duplicates, we screened 14 titles and abstracts and 3 full-texts. Unfortunately, none of the articles was eligible for inclusion. We have updated our flowchart and the manuscript text accordingly.
