# Supplementary material for: Effects of partial penectomy for penile cancer on sexual function: A systematic review
Source: PLoS One. 2022 Sep 22;17(9):e0274914. doi: 10.1371/journal.pone.0274914 (PMC9499284; doi:10.1371/journal.pone.0274914)
Supplement: Attachment — Submitted filename: Response to reviewers.docx [file pone.0274914.s004.docx]

**REVIEWER #2 COMMENT**

**Reviewer comment 1:**

Add discussion about Erectile Dysfunction Inventory of Treatment Satisfaction (EDITS) questionnaires.

**Author response:**

Thank you for this comment and for taking the time to review our manuscript. . We have now included a discussion about the EDITS questionnaire around lines 394-406.
